# Advanced RPL19-TRAP^KI^-seq method reveals mechanism of action of bioactive compounds

**DOI:** 10.1007/s13659-025-00500-3

**Published:** 2025-03-05

**Authors:** Di Zhu, Junchi Hu, Renke Tan, Xiaofeng Lin, Ruina Wang, Junyan Lu, Biao Yu, Yongmei Xie, Xiaohua Ni, Chunmin Liang, Yongjun Dang, Wei Jiang

**Affiliations:** 1 Laboratory of Tumor Immunology, Department of Human Anatomy, Histology and Embryology, School of Basic Medical Sciences, Shanghai Medical College, Fudan University, Shanghai, 200032 China; 2 Basic Medicine Research and Innovation Center for Novel Target and Therapeutic Intervention, Ministry of Education, College of Pharmacy, Chongqing Medical University, Chongqing Medical University, Chongqing, 400016 China; 3 Key Laboratory of Metabolism and Molecular Medicine, Ministry of Education, Department of Biochemistry and Molecular Biology, School of Basic Medical Sciences, Shanghai Medical College, Fudan University, Shanghai, 200032 China; 4 Medical Faculty Heidelberg, Heidelberg University, Heidelberg, Germany; 5 State Key Laboratory of Bioorganic and Natural Products Chemistry, Center for Excellence in Molecular Synthesis, Shanghai Institute of Organic Chemistry, Chinese Academy of Sciences, and University of Chinese Academy of Sciences, Shanghai, 200032 China; 6 State Key Laboratory of Biotherapy and Cancer Center, West China Hospital, Sichuan University and Collaborative Innovation Center of Biotherapy, Chengdu, 610041 China; 7 Shanghai-MOST Key Laboratory of Health and Disease Genomics, NHC Key Lab of Reproduction Regulation, Shanghai Institute for Biomedical and Pharmaceutical Technologies, Shanghai, 200237 China

**Keywords:** TRAP, Ribosome profiling, SBF-1, Oxidative phosphorylation

## Abstract

Natural products play a crucial role in new drug development, but their druggability is often limited by uncertain molecular targets and insufficient research on mechanisms of action. In this study, we developed a new RPL19-TRAP^KI^-seq method, combining CRISPR/Cas9 and TRAP technologies, to investigate these mechanisms. We identified and validated seven ribosomal large subunit surface proteins suitable for TRAP, selecting RPL19 for its high enrichment. We successfully established a stable cell line expressing EGFP-RPL19 using CRISPR knock-in and verified its efficiency and specificity in enriching ribosomes and translating mRNA. Integrated with next-generation sequencing, this method allows precise detection of translating mRNA. We validated RPL19-TRAP^KI^-seq by investigating rapamycin, an mTOR inhibitor, yielding results consistent with previous reports. This optimized TRAP technology provides an accurate representation of translating mRNA, closely reflecting protein expression levels. Furthermore, we investigated SBF-1, a 23-oxa-analog of natural saponin OSW-1 with significant anti-tumor activity but an unclear mechanism. Using RPL19-TRAP^KI^-seq, we found that SBF-1 exerts its cytotoxic effects on tumor cells by disturbing cellular oxidative phosphorylation. In conclusion, our method has been proven to be a promising tool that can reveal the mechanisms of small molecules with greater accuracy, setting the stage for future exploration of small molecules and advancing the fields of pharmacology and therapeutic development.

**Graphical Abstract:**

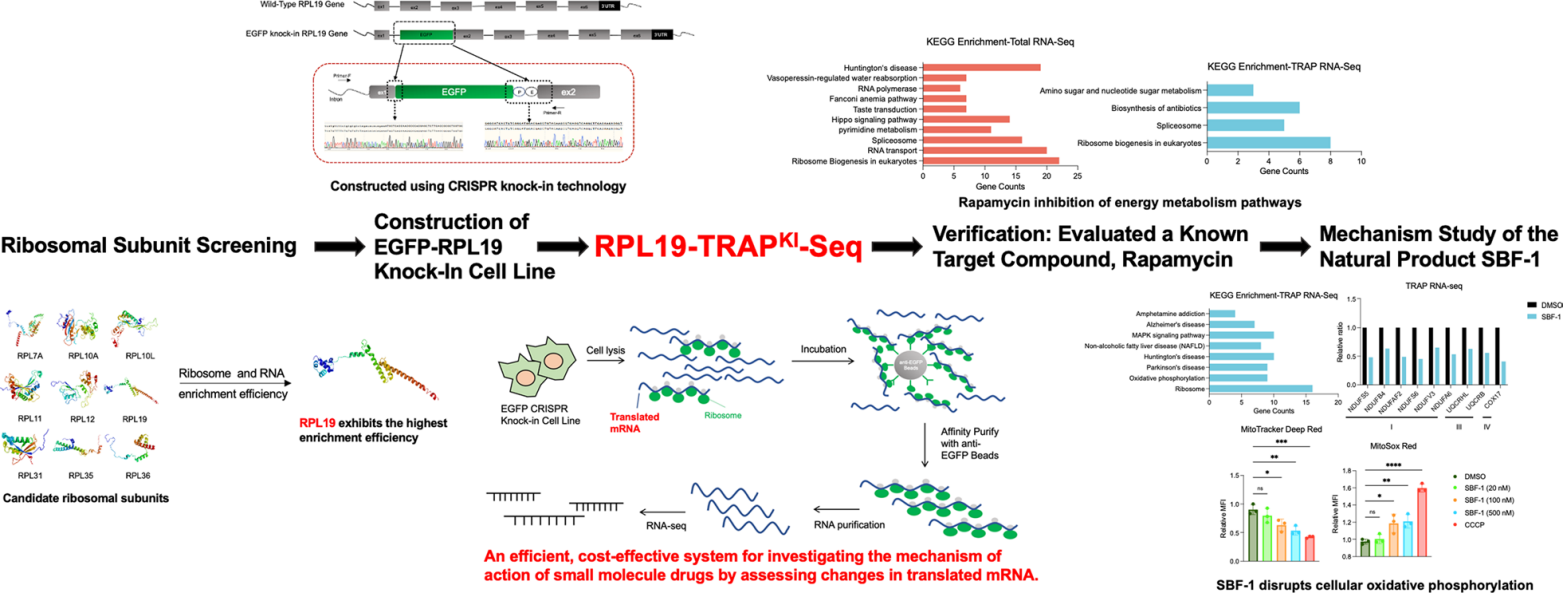

**Supplementary Information:**

The online version contains supplementary material available at 10.1007/s13659-025-00500-3.

## Introduction

In the past few decades, many drugs and new chemical entities (NCEs) have been derived from natural products and their derivatives [1, 2]. Natural products exhibit a broad spectrum of biological activities and play an important role in treating diseases, including cancers [3, 4], acquired immunodeficiency syndrome (AIDS) [5, 6], Alzheimer’s disease [7, 8], malaria [9], pain [10] and so on. Compared with chemically synthesized small molecule drugs, natural products have obvious advantages in structural novelty, biocompatibility, and functional diversity [11]. However, their complex and diverse molecular structures often lead to poor druggability due to potential for non-specific interactions with biomolecules within cells. As a result, natural products require extensive structural modification and optimization during drug development to improve their efficacy and bioavailability. A key bottleneck in this process is the unclear molecular targets and insufficient research on the mechanisms of action. Therefore, target identification and mechanisms of its action are pivotal initial steps toward future drug discovery.

The research on the mechanism of action of natural products faces challenges due to their complex components and multi-target effects, making it difficult for traditional research methods to fully reveal their mechanism. By systematically analyzing genomic, proteomic, and metabolomic data, omics studies can provide multiple layers of biological information to help resolve the complex mechanisms of natural products. These omics-based approaches also enable the identification of potential drug targets and metabolic pathways, thereby enhancing the understanding and application of natural product effects. Several omics-based methods have been developed to study the mechanisms of action of natural products, including Connectivity Map (CMap) [12], Library Integrated Network-Based Cellular Signatures (LINCS) [13], Genome Wide Association Studies (GWAS) [14] and Directionality map (DMAP) [15], and so on. These techniques have been instrumental in elucidating the mechanisms of crucial natural products such as Curcumin [16], Tanshinone IIA [17], Withaferin A [18] and Vincristine [15]. However, each method presents inherent limitations. For instance, the CMap database supports mechanism studies by elucidating correlations between differential gene expression induced by drug treatment and associated diseases [12]. Nevertheless, relying solely on changes in the overall transcriptome of downstream genes may not accurately indicate the specific mechanisms of action of small molecules. Additionally, the number of drugs and cell types included in the CMap database is limited. These constraints limit their applicability, underscoring the need for novel and efficient methods in mechanism research.

Translating ribosome affinity purification (TRAP) employs affinity chromatography to isolate and enrich ribosomes from tissues or cell lysates while capturing the translating mRNA [19]. By overexpressing the EGFP-labeled large ribosomal subunit protein RPL10A in cells or tissues, the labeled RPL10A integrates into ribosome assembly, facilitating ribosome labeling with EGFP [20]. This method, initially applied in neuroscience to identify subtypes of central nervous cells [21, 22], has since been extended to fields such as metabolism, disease, and epigenetics, providing novel solutions to scientific inquiries [23, 24]. Over the past decade, next-generation sequencing (NGS) technology has rapidly developed [25], establishing an efficient sequencing and analysis system alongside advancements in bioinformatics. Integrating TRAP technology with RNA sequencing (RNA-seq) has resulted in TRAP-seq, a novel approach for exploring gene expression at the translational level [26, 27]. Unlike traditional proteomics-based approaches, TRAP-seq provides cost-effective and efficient information through next-generation sequencing, focusing exclusively on actively translating mRNA, closely reflecting true protein expression levels in cells [28]. The application of TRAP technology in drug mechanism studies reduces the non-specific signals of transcriptomic and proteomic methods, revealing the true mechanisms of compounds' action. However, its wide application is currently hindered by its reliance on a limited number of ribosomal subunits, such as RPL10A, for labeling, coupled with low mRNA enrichment efficiency.

In this study, we screened eight ribosomal proteins and verified their efficiency in enriching ribosomal mRNA. We selected the ribosomal large subunit protein RPL19 for its higher enrichment efficiency compared to the traditional RPL10A. Using CRISPR knock-in technology, we integrated an EGFP tag into the RPL19 genome and combined it with NGS to establish an efficient system, RPL19-TRAP^KI^-seq. This system identifies alterations in translating mRNA induced by small molecules, leveraging NGS technology and avoiding excessive reliance on high-cost proteomic approaches. We confirmed the feasibility and reliability of RPL19-TRAP^KI^-Seq technology by verifying the known mTOR inhibitor, rapamycin. Additionally, we conducted a preliminary investigation into the mechanism of SBF-1, a 23-oxa-analog of OSW-1 with potent anti-tumor activity but an unclear mechanism [29], elucidating its potential target range and laying the foundation for subsequent target identification and mechanism exploration.

## Results

### Improved efficiency of ribosome purification with optimized TRAP methods

**Fig. 1:**
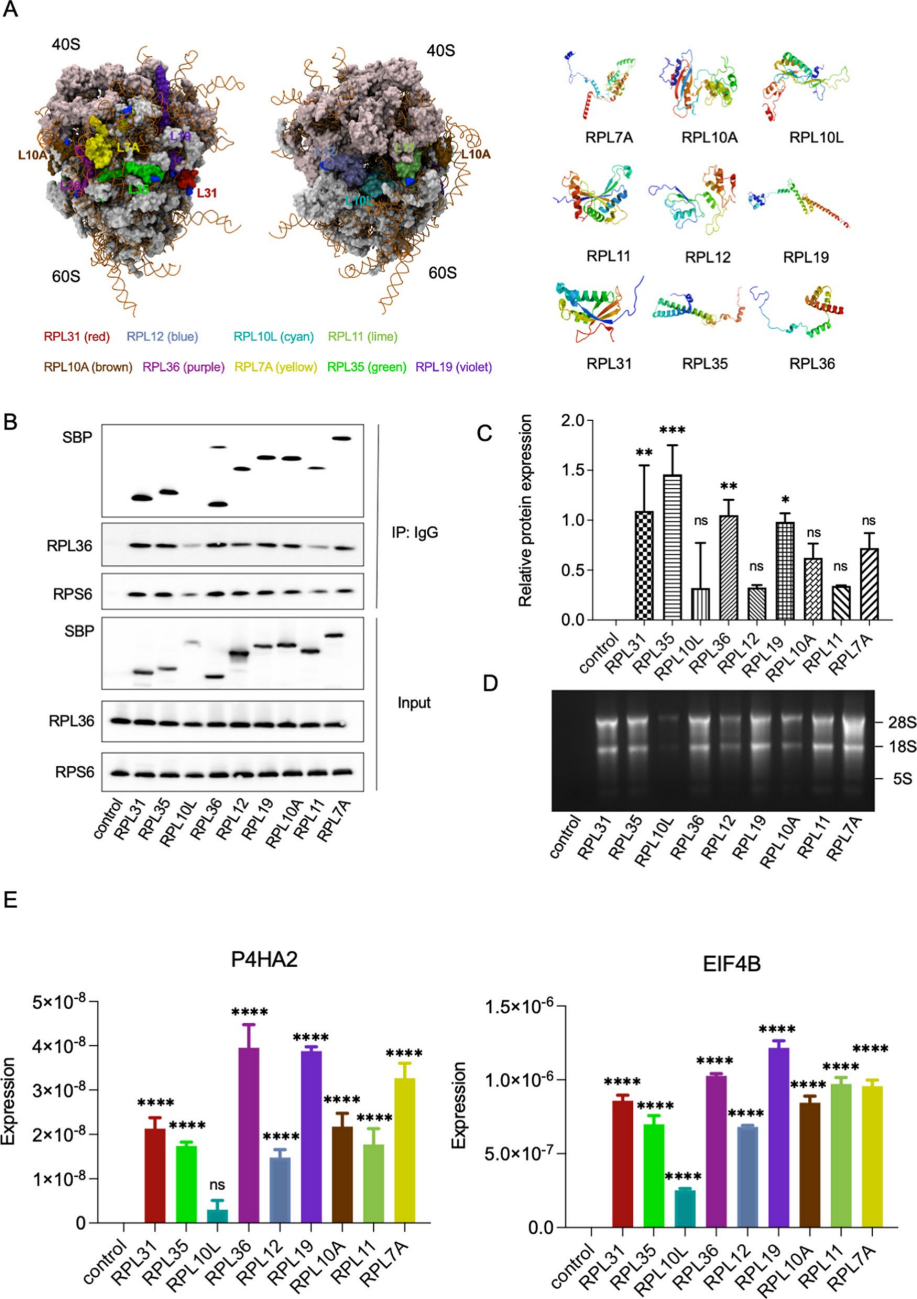
The expression and enrichment efficiency of predicted large ribosomal subunit proteins. **A** Crystal structure of nine human large ribosomal subunit proteins. The N-terminal regions of RPL7A, RPL10A, RPL11, RPL12, RPL19, RPL31, RPL35, and RPL36 are exposed on the ribosomal surface. The N-terminal regions are indicated in blue, and the C-terminal regions are indicated in red. **B**–**E** Plasmids encoding nine predicted large ribosomal subunit proteins were transfected into 293 T cells, followed by collection of cells for endogenous immunoprecipitation as indicated. Cell lysates were used as the input (**B**). Relative protein expression was quantified using ImageJ software, with the ratio of immunoprecipitation signal (SBP of IP) to the input signal (SBP of input) depicted in a bar graph, n = 2 (**C**). RNA content was assessed by agarose gel electrophoresis (**D**). The enrichment of P4HA2 and eIF4B messenger RNA (mRNA) was analyzed by real-time polymerase chain reaction, n = 3 (**E**). Statistical analysis was performed using one-way ANOVA among multiple groups. Bars with asterisks indicate significant differences from the control at ^*^p ≤ 0.05, ^**^p ≤ 0.01, ^***^p ≤ 0.001, ^****^p ≤ 0.0001. Data are represented as mean ± SD

To identify ribosomal proteins suitable for TRAP, we focused on those with exposed N-terminal regions, which facilitate efficient tagging and ribosome purification. We used PyMOL software to analyze the crystal structure of the human 80S ribosome (PDB ID: 4UG0) and predicted eight large subunit proteins that met these criteria: RPL7A, RPL10A, RPL11, RPL12, RPL19, RPL31, RPL35, and RPL36. Among these, RPL10A has been previously reported. Additionally, RPL10L was identified as not being exposed on the ribosomal surface (Fig. 1A). To compare the expression and enrichment efficiency of each subunit, we cloned the nine ribosomal large subunit proteins into vectors and transiently transfected them into HEK293T cells. After immunoprecipitation (IP), we detected both the large subunit protein RPL36 and the small subunit protein RPS6 via Western blotting, indicating successful enrichment of intact ribosomes (Fig. 1B and Figure S1A). Consistent with our predictions, RPL10L, which is not exposed on the ribosomal surface, showed the lowest enrichment level. Quantification of the grayscale intensity of the IP samples and input samples revealed that RPL31, RPL35, RPL36, and RPL19 had pronounced fold changes, meeting the requirements for application (Fig. 1C). To further verify that the large subunit proteins of each ribosome can be separated, we extracted RNA and detected the complete RNA components of the 28S, 18S and 5S (Fig. 1D). Under the same conditions, the total RNA amount enriched by RPL31, RPL35, RPL36, RPL19, RPL11, and RPL7A was significantly pronounced, consistent with the protein levels. We used real-time quantitative PCR (RT-qPCR) to detect the enrichment of *18S*, *GAPDH*, *P4HA2*, and *eIF4B* genes in each sample (Fig. 1E and Figure S1B). These results suggest that RPL19 exhibits the most significant enrichment efficiency and will be used for subsequent experiments.

Thapsigargin is a sesquiterpene lactone isolated from the Mediterranean plant species *Thapsia* [30]. It induces apoptosis in various cell types through Ca^2+^-mediated mitochondrial permeability transition, and disturbances in Ca^2+^ storage and associated signaling can lead to endoplasmic reticulum (ER) stress [31]. The transcription factors CHOP and ATF4 are elevated in response to ER stress [32, 33]. To verify the optimization effect of TRAP method, we transfected RPL19 into HEK293T cells for 24 h, treated the cells with 1 μM thapsigargin for 10 h, and utilized TRAP to collect samples for detecting RNA alterations. As expected, protein levels of ATF4 and CHOP were significantly elevated (Figure S1C). In cell lysates, RNA levels of *ATF4* increased by twofold, while RNA levels of *CHOP* increased by fivefold. However, in TRAP affinity-purified RNA, the *ATF4* levels increased by twofold, while the *CHOP* levels increased by sevenfold (Figure S1D). These results strongly suggest that TRAP is more sensitive and rapid in detecting compound-induced RNA changes during a specific time period compared to total RNA.

### Successful establishment of EGFP-RPL19 knock-in cell line using CRISPR technology

**Fig. 2:**
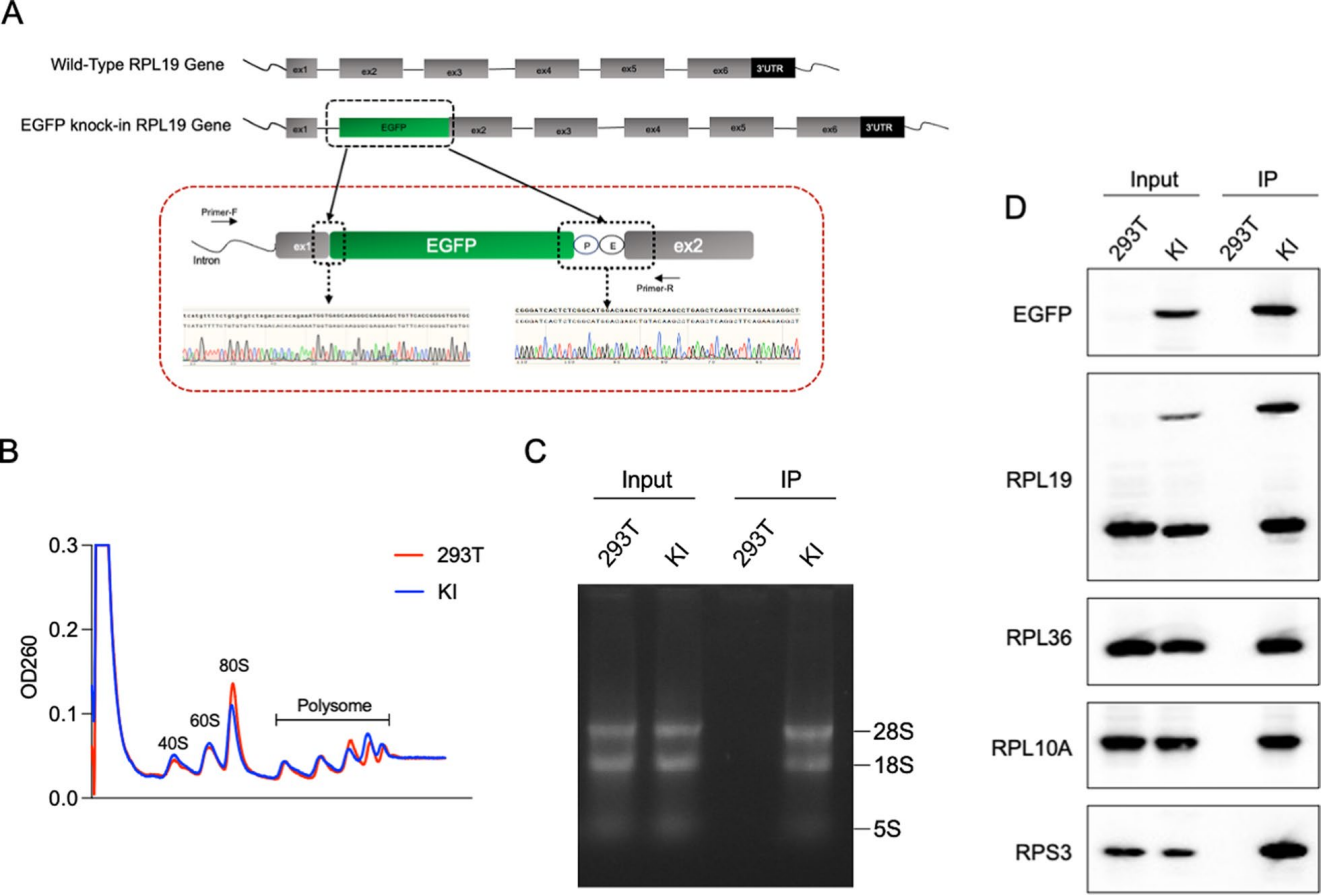
Establishment of the EGFP-RPL19 cell line using CRISPR knock-in technology. **A** Schematic representation of the experimental setup of constructing a cell line stably expressing EGFP-RPL19. **B** Polyribosome distribution of EGFP-RPL19 knock-in cell line (blue) and HEK293T WT cells (red). **C**, **D** Cell lysates from HEK293T and EGFP-RPL19 knock-in cells were immunoprecipitated using EGFP affinity beads. RNA was extracted from the cell lysates and the immunoprecipitated products. RNA content was assessed by agarose gel electrophoresis (**C**), and the expression of ribosomal proteins RPS3, RPL10A, RPL36, RPL19, and EGFP was analyzed by western blotting (**D**)

To overcome the limitations of exogenous expression, we constructed stable cell lines using CRISPR knock-in technology [34]. The first exon of RPL19 contains a single start codon ATG, so we inserted an enhanced green fluorescent protein (EGFP) into the N-terminus of the second exon of RPL19, which was verified by PCR (Fig. 2A). After monoclonal screening and culture, we observed that the majority of green fluorescence was distributed in the cytoplasm, consistent with the typical ribosomal distribution (Figure S2A). PCR analysis of three monoclonal cell lines showed consistent bands at 1000 bp in all samples, confirming the successful insertion of the *EGFP* gene. The presence of two bands in each sample indicated heterozygosity (Figure S2B). Western blotting further confirmed successful insertion of the EGFP gene (Figure S2C). Among these, Clone #1 was selected for further investigation due to its high expression of the EGFP-RPL19 fusion protein.

Next, we assessed whether the cell line could reliably and selectively enrich ribosomes and mRNA. The polysome profiling assay was used to separate all ribosomal components within cells. The results showed that the distributions of ribosomal components in the knock-in cells and the HEK293T wild-type (WT) cells were fundamentally consistent, particularly at the 40S, 60S, and 80S positions, where they almost completely overlapped (Fig. 2B). This indicates that the insertion of the *EGFP* gene does not impact the overall translation level of the cell. We then conducted an IP assay to assess the specificity and efficiency of ribosome enrichment in the EGFP-RPL19 knock-in cells. Agarose gel electrophoresis demonstrated that this cell line can effectively and selectively enrich RNA (Fig. 2C). Furthermore, western blotting analysis revealed that the 50 kDa EGFP-RPL19 fusion protein, free RPL19 protein, and other ribosomal proteins (RPL36, RPL10A, and RPS3) were specifically enriched in the knock-in cells, but not in the HEK293T WT cells, underscoring the specificity of ribosomal protein enrichment in the EGFP-RPL19 cells (Fig. 2D). The specificity was further confirmed by silver staining experiments (Figure S2D). In summary, we have successfully established an EGFP-RPL19 knock-in cell line using CRISPR technology, addressing the challenges of non-specific enrichment and complex background commonly associated with the TRAP method. This cell line reliably enriches ribosomes and mRNA, maintaining consistent ribosomal component distribution and specific protein enrichment.

### Feasibility of RPL19-TRAPKI-Seq in drug mechanism research

**Fig. 3:**
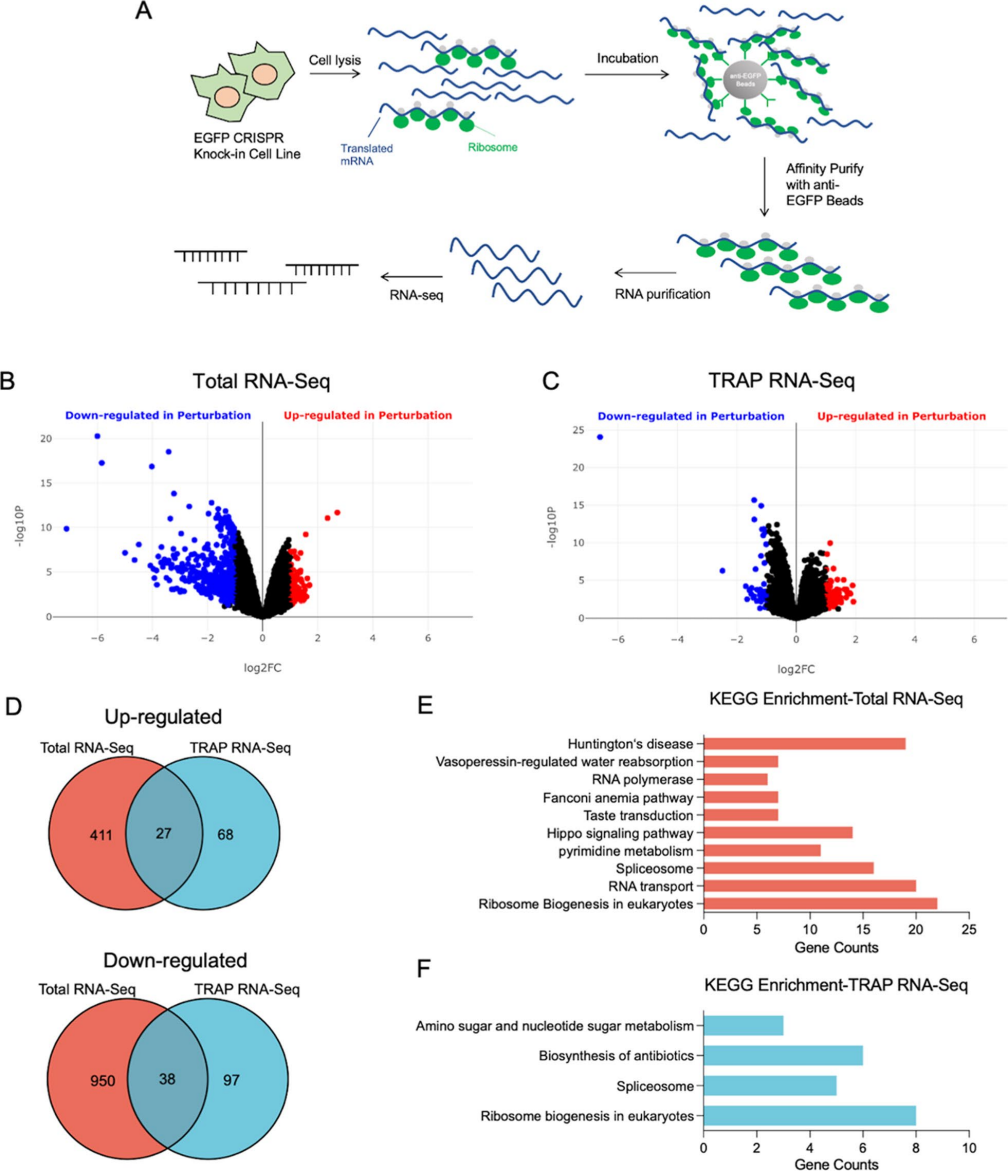
RPL19-TRAP^KI^-Seq for studying the mechanism of action of small molecules. **A** Flowchart of RPL19-TRAP^KI^-Seq to investigate the mechanism of action of active small molecule drugs. **B**, **C** Volcano plots of the up- and down-regulated genes in total RNA (**B**) and TRAP RNA (**C**) after rapamycin treatment. **D** Venn diagram illustrating the number of up- and down-regulated genes in total RNA and TRAP RNA after rapamycin treatment. **E**, **F** Differentially expressed genes of total RNA (**E**) and TRAP RNA (**F**) were enriched using KEGG pathways, and the number of genes was counted and represented in a bar graph. KEGG, Kyoto Encyclopedia of Genes and Genomes

RNA-Seq is a powerful method for transcriptome profiling using deep-sequencing technologies. Our previous results demonstrated that the EGFP-RPL19 knock-in cell line functions as an optimized TRAP system for ribosome purification. We hypothesized that integrating this optimized TRAP method with RNA-Seq (RPL19-TRAP^KI^-Seq) would expedite mechanism elucidation. As shown in the flowchart, TRAP is employed to purify the translating mRNA, which is then sequenced to identify the mechanism (Fig. 3A). To investigate this hypothesis, we used rapamycin, a macrolide compound, known to inhibit translation and induce autophagy by targeting mTOR [35–37]. We treated EGFP-RPL19 knock-in cells with 100 nM rapamycin for 6 h. Then, we lysed the cells and extracted total mRNA from one portion of the supernatant, while enriching ribosome-bound mRNA using EGFP beads from another portion to obtain TRAP mRNA. RNA-Seq analysis generated volcano plots for both total mRNA and TRAP mRNA (Fig. 3B, C). At the total mRNA level, 437 genes were up-regulated and 988 were down-regulated. For TRAP mRNA, 94 genes were up-regulated and 134 were down-regulated, with 27 genes up-regulated and 38 down-regulated in both datasets (Fig. 3D).

KEGG enrichment analysis of differentially expressed genes revealed that rapamycin treatment affects multiple pathways, including ribosome biogenesis, RNA transport, Huntington's disease, Hippo signaling, and the spliceosome pathway (Fig. 3E). TRAP mRNA enrichment data showed differentially expressed genes clustered in four pathways: ribosome biogenesis, amino sugar and nucleotide sugar metabolism, biosynthesis of antibiotics, and spliceosomes pathways (Fig. 3F). Downregulation in 'ribosome biogenesis' and 'spliceosomes' clearly indicates rapamycin’s inhibitory effect on translation[38, 39]. The overlapping genes in 'biosynthesis of antibiotics' and 'amino sugar and nucleotide sugar metabolism' include enzymes related to glucose and energy metabolism, indicating inhibition of the energy metabolism pathway, a critical component of mTOR function. These findings confirm that mTOR is the endogenous target of rapamycin. Thus, we have established RPL19-TRAP^KI^-Seq as a novel, efficient, and cost-effective system that minimizes background interference in small molecule drug mechanism research, verified by rapamycin.

### RPL19-TRAP^KI^-Seq reveals disruption of oxidative phosphorylation by the antitumor compound SBF-1

**Fig. 4:**
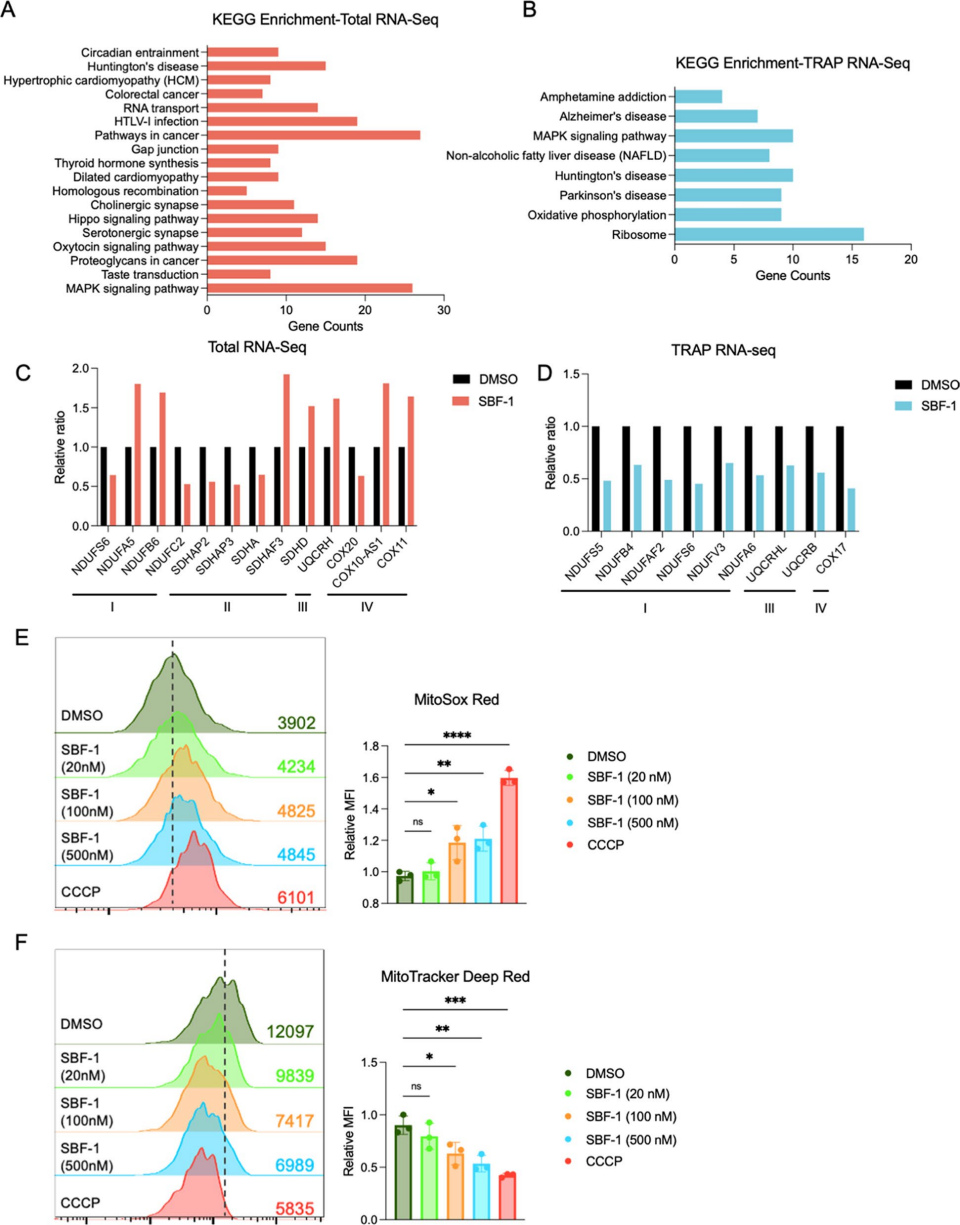
SBF-1 disrupts cellular oxidative phosphorylation. **A**, **B** Differentially expressed genes in total RNA (**A**) and TRAP RNA (**B**) after SBF-1 treatment were enriched using KEGG pathways, and the number of genes is represented in a bar graph. **C**, **D** Relative ratios of mitochondria-related differentially expressed genes in total RNA (**C**) and TRAP RNA (**D**) after SBF-1 treatment, based on RNA-sequencing data. **E**, **F** Representative histograms (left) and quantifications (right) of the mean fluorescence intensity (MFI) of MitoSOX Red (**E**) and MitoTracker™ Deep Red (**F**) at different concentrations of SBF-1, n = 3. Numbers in graphs indicate the mean fluorescence intensity (MFI). Statistical analysis was performed using one-way ANOVA among multiple groups. Bars with asterisks indicate significant differences from the control at ^*^p ≤ 0.05, ^**^p ≤ 0.01, ^***^p ≤ 0.001. Data are represented as mean ± SD

OSW-1, a steroidal saponin from O. saundersiae [40], exhibits potent cytotoxicity, outperforming clinical anti-cancer drugs such as etoposide, doxorubicin and methotrexate [41, 42]. Its analog, SBF-1, shows similar or superior antitumor activity and is easier to synthesize [43]. We employed RPL19-TRAP^KI^-Seq to investigate SBF-1’s mechanism of action. In EGFP-RPL19 knock-in cells, the IC_50_ value of SBF-1 was 1.763 nM (Figure S3B). Treatment with 20 nM SBF-1 for 6 h (Figure S3C, S3D) revealed 339 up-regulated and 943 down-regulated genes in total mRNA. Regarding TRAP mRNA, 32 genes were up-regulated and 186 genes were down-regulated, with 20 genes up-regulated and 49 down-regulated in both datasets (Figure S3E). KEGG enrichment analysis of TRAP mRNA changes (Fig. 4A) identified eight pathways: Ribosome, Oxidative phosphorylation, Parkinson's disease, Huntington's disease, Alzheimer's disease, MAPK signaling pathway, NAFLD, and Amphetamine addiction (Fig. 4B). Differentially expressed genes in these pathways indicated that SBF-1 affects protein complexes I/III in oxidative phosphorylation, corroborated by consistent down-regulation of mitochondria-related genes in TRAP mRNA (Fig. 4C, D). Given that oxidative phosphorylation is a critical process for cellular energy production and that its disruption is known to induce cell death, particularly in cancer cells, we hypothesized that SBF-1 exerts its cytotoxic effects by targeting this pathway. Further analysis using MitoSox Red and MitoTracker Deep Red showed that increasing SBF-1 concentrations significantly reduced mitochondrial oxygen consumption and membrane potential (Fig. 4E, F). These results demonstrate that SBF-1 induces cell death by disrupting oxidative phosphorylation and inhibiting mitochondrial respiration.

## Discussion

This study demonstrates the successful optimization and application of the RPL19-TRAP^KI^-Seq system for compounds mechanism research, particularly focusing on the anti-tumor compound SBF-1. TRAP technology has become a potent tool for exploring expression profiles across various research fields [44–46]. In this study, we improved the TRAP system by selecting the RPL19 ribosomal protein, which has greater enrichment efficiency compared to the traditional RPL10A subunit, and by establishing an EGFP-RPL19 knock-in cell line, thereby significantly enhancing the efficiency and stability of mRNA enrichment.

Utilizing the RPL19-TRAP^KI^-Seq method, we confirmed the mechanism of action of rapamycin. Compared to transcriptome RNA sequencing, TRAP RNA sequencing demonstrated superior specificity, accurately highlighting the inhibition of translation and energy metabolism pathways by rapamycin. The observed changes in gene expression levels clearly indicated that rapamycin's endogenous target is mTOR. These findings validated the feasibility and specificity of our optimized TRAP system for identifying drug targets and elucidating mechanisms of action. We then applied this optimized system to investigate the mechanism of SBF-1, a 23-oxa-analog of OSW-1 known for its potent anti-tumor activity. It has been reported that Oxysterol binding protein (OSBP) and OSBP-related protein 4L (ORP4L) can directly bind OSW-1, however, both genes are not directly related to cell growth and apoptosis. Furthermore, the down-regulation of about 85% OSBP protein expression does not affect cell growth or induce apoptosis [47]. Therefore, the authentic mechanism of OSW-1 in inhibiting cell growth and inducing apoptosis needs further exploration. Despite the challenges associated with the unclear mechanism of action of OSW-1, our study narrowed down the mechanism of SBF-1 to its impact on intracellular mitochondrial oxidative phosphorylation. RNA-Seq analysis revealed significant down-regulation of mitochondria-related genes and pathways involved in oxidative phosphorylation. Consistent with these findings, flow cytometry demonstrated that SBF-1 significantly reduces mitochondrial oxygen consumption and membrane potential, suggesting a disruption in mitochondrial respiration as the primary mode of action.

The TRAP method was optimized and applied for the first time in drug mechanism research, offering specific advantages. Enhanced enrichment efficiency enables the acquisition of sufficient mRNA from a small number of cells, in contrast to larger sample volumes required for mass spectrometry-based methods. This innovation facilitates the investigation of mechanisms involving low-abundance, complex-to-isolate, and challenging-to-synthesize natural products. Additionally, TRAP technology provides shorter processing times and lower costs for mechanism research. While the RPL19-TRAP^KI^-Seq method may not directly identify compound targets, the mRNA enrichment achieved through TRAP correlates closely with changes in protein levels, offering a more focused impact than transcriptome sequencing alone. In the future, generating knock-in mice may become a viable option for in vivo studies of drug mechanisms.

While the RPL19-TRAPKI-Seq system offers significant advantages, it also has several limitations that should be considered. First, although the method significantly improves mRNA enrichment efficiency, it remains biased towards ribosome-bound mRNA, potentially excluding non-ribosome-associated transcripts that may play crucial roles in understanding the full range of compound effects. Second, despite its superior specificity in identifying rapamycin's effects compared to traditional transcriptome RNA sequencing, the method can’t directly identify drug targets, which remains a significant drawback. Furthermore, the system's reliance on mRNA levels as proxies for protein activity may not always be accurate, as mRNA translation is affected by various regulatory mechanisms not captured by this method. Therefore, integrating TRAP technology with complementary target identification techniques could enhance the investigation of active small molecule mechanisms and aid in drug development.

In conclusion, our study demonstrates the significant potential of the RPL19-TRAP^KI^-Seq system in compound mechanism research, providing a new, efficient, and cost-effective approach for target identification and mechanism research. These findings lay the groundwork for future research into the mechanisms of action of small molecules like SBF-1 and beyond. By integrating TRAP technology with advanced sequencing techniques, we have established an efficient platform that can contribute to the field of pharmacology and therapeutic development.

## Materials and methods

### Cell culture

HEK293T cells preserved in our laboratory were maintained in Dulbecco’s Modified Eagle Medium (DMEM, Gibco). HAP1 cells were maintained in Iscove's Modified Dulbecco's Medium (IMDM, Gibco). All media were supplemented with 10% fetal bovine serum (FBS, Gibco) and 100 U/ml penicillin and 100 μg/ml streptomycin, and cells were maintained at 37 °C in 5% CO_2_.

### Structure solution by PyMOL

A cryo-electron microscopy structure of the human 80S ribosome (PDB ID: 4UG0) was utilized to identify potentially modifiable subunits. The 3D structure of the human 80S ribosome was visualized using PyMOL software. Effective marker proteins can only be provided by subunits exposed on the surface of the ribosome. Therefore, we selected eight subunits with exposed N-termini: RPL7A, RPL10A, RPL11, RPL12, RPL19, RPL31, RPL35, and RPL36.

### Gateway

The Gateway™ compatible vectors pcDNA5/(RPL7A), pcDNA5/(RPL10A), pcDNA5/(RPL10L), pcDNA5/(RPL11), pcDNA5/(RPL12), pcDNA5/(RPL19), pcDNA5/(RPL31), pcDNA5/(RPL35), and pcDNA5/(RPL36) were cloned into pDONR™ (Invitrogen) using the Gateway entry clone system (Invitrogen). These entry vectors were then combined with pcDNA5/FRT/TO carrying the STAP tag using the LR Clonase™ enzyme (Invitrogen). After a 1-h incubation at 25 °C and treatment with Proteinase K for 10 min, the reaction mixture was used for the transformation of *DH5α E. coli* cells, and the plasmids were extracted using a plasmid DNA extraction kit (TIANGEN).

### Translating ribosome affinity purification (TRAP)

2 × 10^6 HEK293T cells were seeded in 60-mm-diameter tissue culture dishes until the cell density reached 70–80%. Transfection of plasmids was performed using PEI transfection reagent. After 24 h, cells were treated with 100 μg/ml cycloheximide (CHX) (Sigma) for 15 min, then collected and washed 3 times with PBS containing 100 μg/mL CHX. The cell pellets were resuspended in TMK100 lysis buffer (10 mM Tris–HCl, pH7.4; 100 mM KCl; 5 mM MgCl_2_; 1% Triton X-100; 2 mM DTT) with 1 mM PMSF, protease inhibitor cocktail (Sigma), 100 μg/ml cycloheximide, and 10 μl/ml RNasin RNase inhibitors (Promega), and incubated on ice for 15 min. Lysates were cleared by centrifugation at 12,000 rpm for 5 min at 4 °C. 40 μl of the supernatants were transferred to a new 1.5 mL microcentrifuge tube containing 20 μl IgG Beads (Invitrogen), incubated with gentle rotation for 3 h at 4 °C, then centrifuged at 3,000 rpm for 2 min at 4 °C. 30 μl of the supernatants were washed three times with TMK100 lysis buffer and once with SBP solution (10 mM Tris–HCl, pH7.5; 100 mM NaCl; 0.2% NP-40). To remove the Protein A tag, IgG beads were incubated in 30 μl SBP solution with 3 μl AcTEV enzyme (Thermo) and digested at 16℃ for 2 h with shaking and mixing every 30 min. After centrifugation, the supernatant was transferred to a new 1.5 mL microcentrifuge tube and boiled with 6 × SDS sample buffer for 5 min to be used as IP samples.

### Western blotting

Cells were washed with PBS, then lysed in RIPA lysis buffer (Sigma) containing a protease inhibitor cocktail (Sigma) and 1 mM PMSF. Protein concentration was measured using the BCA protein assay kit (Sigma). The samples were then loaded onto 10% gels along with a prestained protein ladder (Invitrogen) for electrophoresis. Proteins were transferred to PVDF membranes (Amersham) and blocked with a 5% (w/v) non-fat dry milk solution at room temperature for 1 h. Primary antibodies were incubated at 4 ℃ overnight. The membranes were then incubated with HRP-conjugated secondary antibodies at room temperature for 1 h, followed by washing with PBST. The target proteins were visualized using the ECL detection system (Clinx Science Instruments Co. Ltd) and analyzed with ImageJ. The following antibodies were used: anti-SBP, anti-RP19, anti-RPS3, anti-RPL10A, and anti-GAPDH (Santa Cruz Biotechnology); anti-RPL36 (Epitomics), anti-RPS6 (Clontech), anti-EGFP (Abway) and HRP-conjugated secondary antibodies (Calbiochem).

### Real-time quantitative PCR (RT-qPCR)

Total RNA was extracted using the TRIzol Kit (Invitrogen). cDNA was synthesized using Reverse Transcription Kit (Takara). Real-time PCR was performed with SYBR Green PCR mix (Roche), using the following primers: 18 S, 5′-AAACGGCTACCACATCCAAG-3′ (forward) and 5′-CCTCCAATGGATCCTCGTTA-3′ (reverse); eIF4B, 5′-TTTCCCTCTCCCAACATGG-3′ (forward) and 5′-GTGCTTCCTCCACCAGTACC-3′ (reverse); P4HA2, 5′-GGTGAAGCGGCTAAACACAGAC-3′ (forward) and 5′-CAGTCATCCACACTCAGCATTGC-3′ (reverse); GAPDH, 5′-GAGCCTCAAGATCATCAGCA-3′ (forward) and 5′-ACAGTCTTCTGGGTGGCAGT-3′ (reverse).

### Construction of CRISPR knock-in cells

EGFP-RPL19 knock-in HEK293T cells were generated using the CRISPR/Cas9 system. The guide RNA (sgRNA) sequences used were: 5′-CACCGtctgtgtgtctagTATGCTC-3′ (forward) and 5′-CAAAGAGCATActagacacacagaC-3′ (reverse). These sgRNAs were amplified and ligated into the LentiCRISPR VII vector. The EGFP encoding fragment was inserted into the Puc19 vector via homologous recombination. The primers used for this process were: Puc19-EGFP, 5′-tcacacaggaaacagctatgacCCTGAGCATActagacacacagaaATGG-3′ (forward) and 5′-gtaaaacgacggccagtgaattctctgtgtgtctagTATGCTCAGGCTTGT-3′ (reverse); EGFP TEST, 5′-gtcttgatggggacgttcatt-3′ (forward) and 5′-GAGTTGGCATTGGCGATTTC-3′ (reverse). *Escherichia coli* transformation and plasmid isolation were performed according to standard protocols. HEK293T cells were transfected with the lentiCRISPR-sgRPL19 plasmid and Puc19-EGFP plasmid. After incubation for 48 to 96 h, EGFP-expressing cells were selected using a fluorescence microscope (Olympus) and cultured to generate monoclonal cell lines. Single clones were validated by Western blotting, PCR and nucleotide sequencing.

### Cell cytotoxicity assay

2 × 10^3^ cells were seeded into 96-well plates and incubated with indicated concentrations of SBF-1 at 37 °C for 72 h. Cell viability was assessed using the CellTiter-Glo assay (Promega) following the manufacturer’s protocol.

### Silver staining

After proteins were separated by SDS-PAGE, the gels were fixed (50% MeOH; 12% Acetic acid; 1.85% formaldehyde) at room temperature for 1 h or overnight at 4 ℃. The gels were rinsed three times in 50% MeOH/water for 20 min each and washed in deionized water. Following a 1-min soak in sodium thiosulfate at room temperature, the gels were stained with 0.2% silver nitrate for 20 min until sufficiently dark. The staining reaction was stopped with 50% MeOH and 12% acetic acid.

### Polysome profiling

Transfected HEK293T cells were treated with 100 μg/ml cycloheximide (CHX) (Sigma) for 15 min, then washed twice with PBS containing 100 μg/ml CHX. The cells were lysed in 500 μl TMK100 lysis buffer (containing RNase and protease inhibitor) on ice for 5 min. The lysate was centrifuged at 12,000 rpm for 10 min at 4 °C. RNA concentrations of the supernatants were measured by a NanoDrop instrument (Thermo). 300 μl of normalized supernatants were added to prepared 15% and 45% sucrose density gradients (containing RNase and protease inhibitor) and fractionated in an SW41 Ti rotor (Beckman) at 38,000 rpm for 3 h at 4 ℃. Polysome fractions were monitored using a gradient preparation and fractionation system (BioComp). RNase-free reagents and filter-tip pipettes were used throughout the procedure.

### RNAseq

For the purification of translated RNA following treatment with rapamycin (Selleck Chemicals LLC) and SBF-1 (a kind gift from Prof. Biao Yu at Shanghai Institute of Organic Chemistry), EGFP-RPL19 knock-in cells were treated with 100 nM rapamycin and 20 nM SBF-1 for 6 h, followed by treatment with 100 μg/ml CHX for 15 min before lysis. Cells were washed twice with 4 ml of ice-cold PBS (containing 100 μg/ml CHX) and immediately homogenized in 1 ml ice-cold low salt wash buffer. Cell lysate was centrifuged at 12,000 rpm for 15 min at 4 °C. The supernatant was retained and incubated with GFP antibody-labeled agarose resin for 1 h end-over-end rotation at 4 °C. Beads were subsequently collected by centrifugation and washed four times with high salt wash buffer. RNA was extracted using the Absolutely RNA Nanoprep kit (Agilent) according to the manufacturer′s instructions. Libraries were prepared using the NEB Next® Ultra™ Directional RNA Library Prep Kit for Illumina (New England Biolabs) and sequenced on the Illumina HiseqTM4000 platform.

### Flow cytometry

HAP1 cells in the logarithmic growth phase were seeded in 96-well plates at a density of 7 × 10^4^ cells/well. DMSO control or SBF-1 (20 nM, 100 nM, 500 nM) was added to each well and incubated at 37 °C for 6 h. Carbonyl cyanide m-chlorophenyl hydrazone (CCCP, Selleck) was used as a positive control to treat cells for 30 min. Dead cells were excluded by Fixable Viability Dye eFluor™ 780 (eBioscience). For detection of cellular ROS levels, cells were stained with 5 μM MitoSox Red (Thermo) in HBSS for 15 min at 37 °C. For analysis of mitochondrial membrane potential, MitoTracker Deep Red (Thermo) staining was used at 5 nM in HBSS for 30 min at 37 °C. Cells were then washed and resuspended in HBSS for FACS analysis. Flow cytometry (Beckman CytoFLEX S) was used according to the instructions, and FlowJo was used to analyze the results.

### Statistical analysis

Experimental data were analyzed using GraphPad Prism version 9. P values were calculated using ordinary one-way ANOVA.

## Supplementary Information

Supplementary Material 1

## Data Availability

Data will be provided upon request.

## Abbreviations

CMap: Connectivity Map
LINCS: Library Integrated Network-Based Cellular Signatures
GWAS: Genome Wide Association Studies
DMAP: Directionality Map
TRAP: Translating Ribosome Affinity Purification
NGS: Next-Generation Sequencing
RNA-seq: RNA Sequencing
IP: Immunoprecipitation
RT-qPCR: Real-Time Quantitative PCR
ER: Endoplasmic Reticulum
EGFP: Enhanced Green Fluorescent Protein
WT: Wild-Type
OSBP: Oxysterol Binding Protein
ORP4L: OSBP-Related Protein 4L
CHX: Cycloheximide
mRNA: Messenger RNA
MFI: Mean Fluorescence Intensity
