# Supplementary material for: Advanced RPL19-TRAPKI-seq method reveals mechanism of action of bioactive compounds
Source: Nat Prod Bioprospect. 2025 Mar 5;15(1):16. doi: 10.1007/s13659-025-00500-3 (PMC11882491; doi:10.1007/s13659-025-00500-3)
Supplement: Supplementary file 1 — Supplementary Material 1 [file 13659_2025_500_MOESM1_ESM.docx]

Figure S1


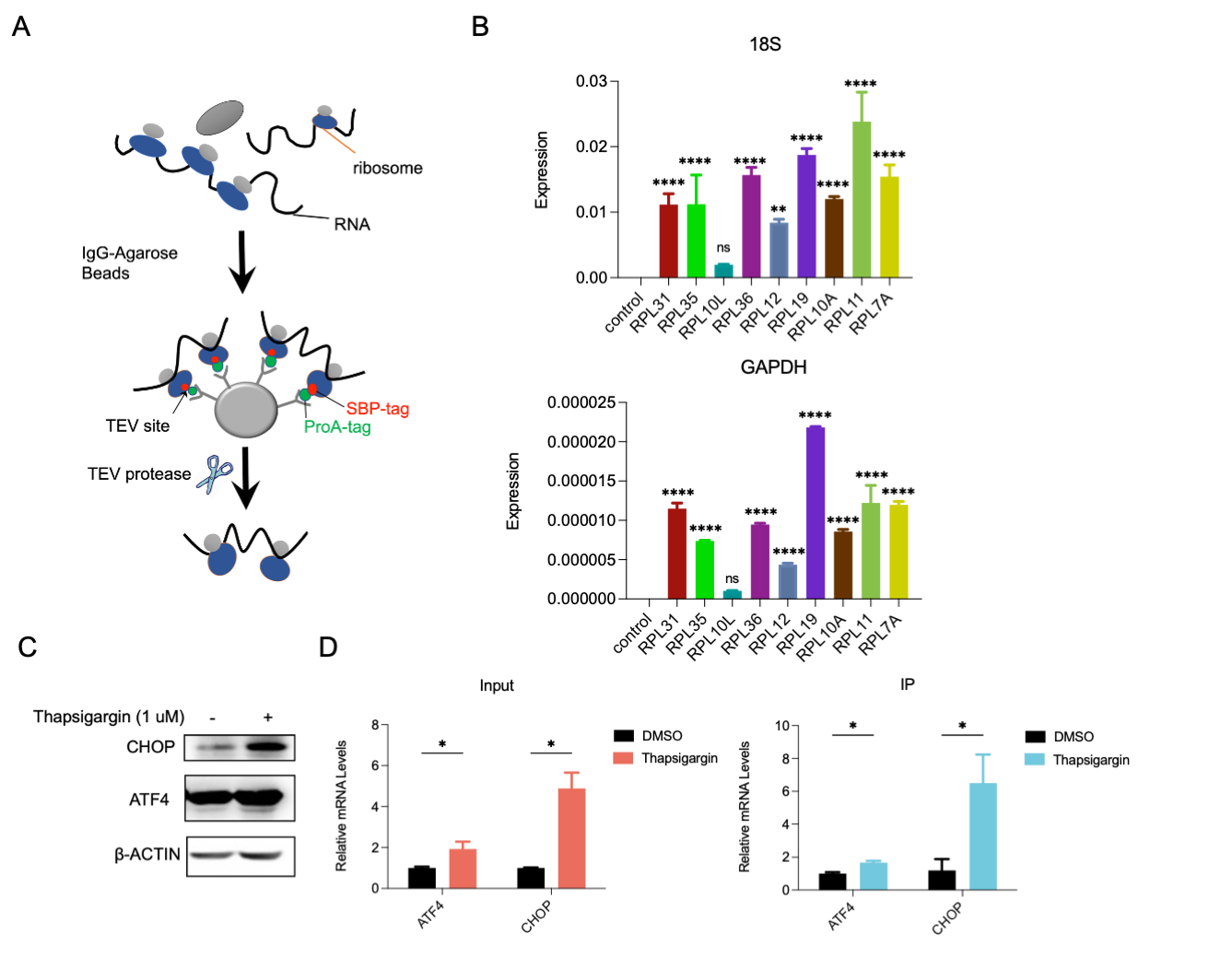


**Figure S1. TRAP is more sensitive and rapid in detecting drug-induced RNA changes**

(A) Schematic of the experimental principle of ribosome affinity purification. Exogenous ProA-TEV-SBP-RPL complex is expressed and integrated into the ribosome complex. IgG beads initially bind ribosome complex with exogenous protein. After TEV enzyme cleavage, the ribosome-RNA complex can be isolated for subsequent analysis.

(B) Enrichment analysis of 18S rRNA and GAPDH by real-time PCR. Statistical analysis was performed using one-way ANOVA among multiple groups. Bars with asterisks indicate significant differences from the control at ^*^p ≤ 0.05, ^**^p ≤ 0.01, ^***^p ≤ 0.001, ^****^p ≤ 0.0001. Data are represented as mean ± SD.

(C) Western blotting analysis of the expression of ATF4 and CHOP in the presence or absence of 1 μM Thapsigargin.

(D) Real-time PCR analysis of ATF4 and CHOP in cell lysates (Input) and TRAP immunoprecipitation samples (IP). Statistical analysis was performed using Multiple unpaired t tests. Bars with asterisks indicate significant differences from the control at ^*^p ≤ 0.05, ^**^p ≤ 0.01, ^***^p ≤ 0.001, ^****^p ≤ 0.0001. Data are represented as mean ± SD.

Figure S2


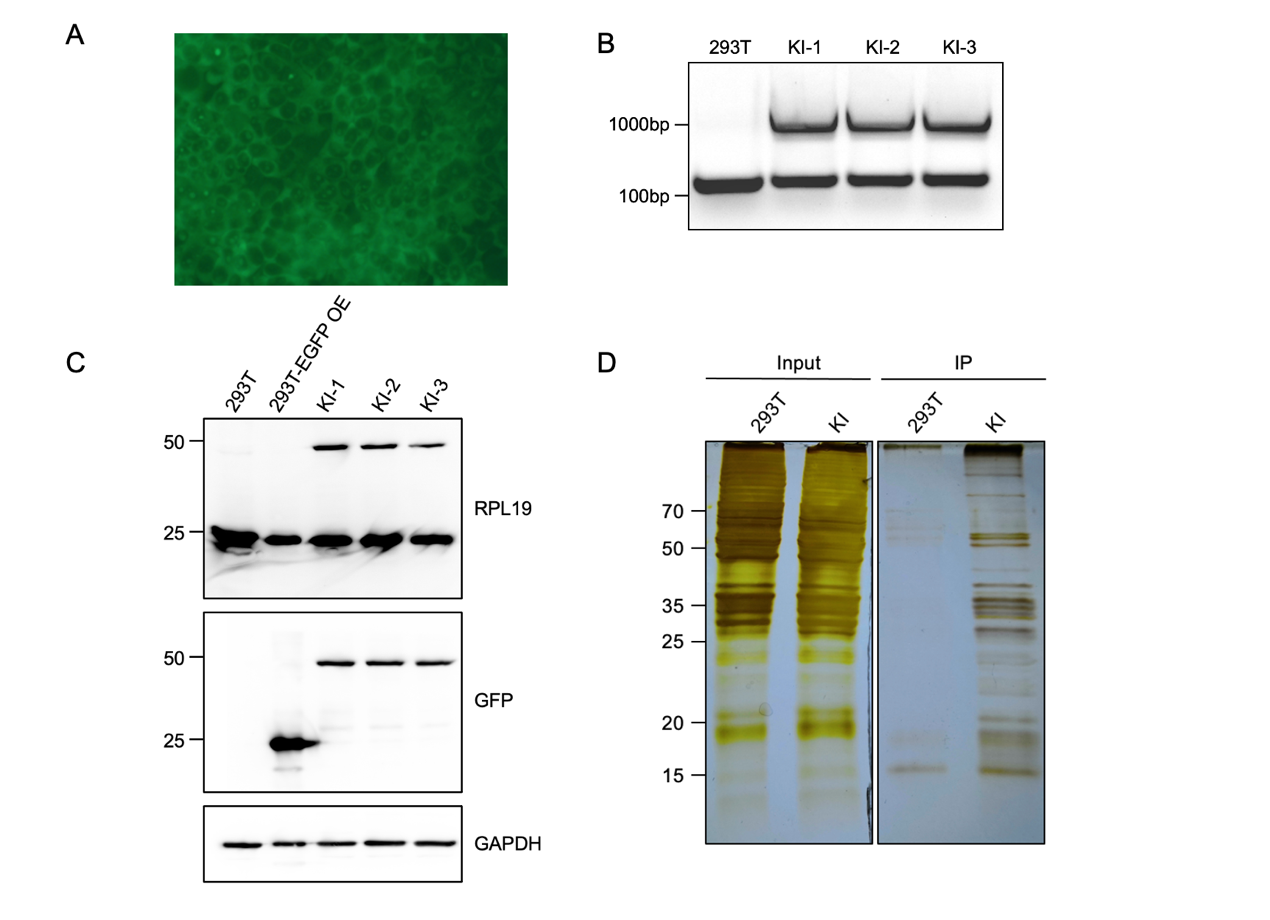


**Figure S2. Establishment of the EGFP-RPL19 cell line**

(A) Fluorescence microscopy image of the cell line stably expressing EGFP-RPL19.

(B) PCR amplification of the EGFP insertion fragment using the genome of EGFP knock-in cells or HEK293T WT cells as templates. The inserted EGFP fragment band is located around 1000 bp, while the band at 200 bp indicates the uninserted fragment.

(C) Western blotting analysis of the expression of RPL19 and GFP. Protein lysates from HEK293T WT cells (panel 1), HEK293T cells transiently transfected with EGFP (panel 2) and EGFP knock-in cells (panels 3-5) were separated by SDS-PAGE gel electrophoresis.

(D) Immunoprecipitation of HEK293T and EGFP-RPL19 knock-in cell lysates using EGFP affinity beads. The immunoprecipitated proteins were detected by silver staining.

Figure S3


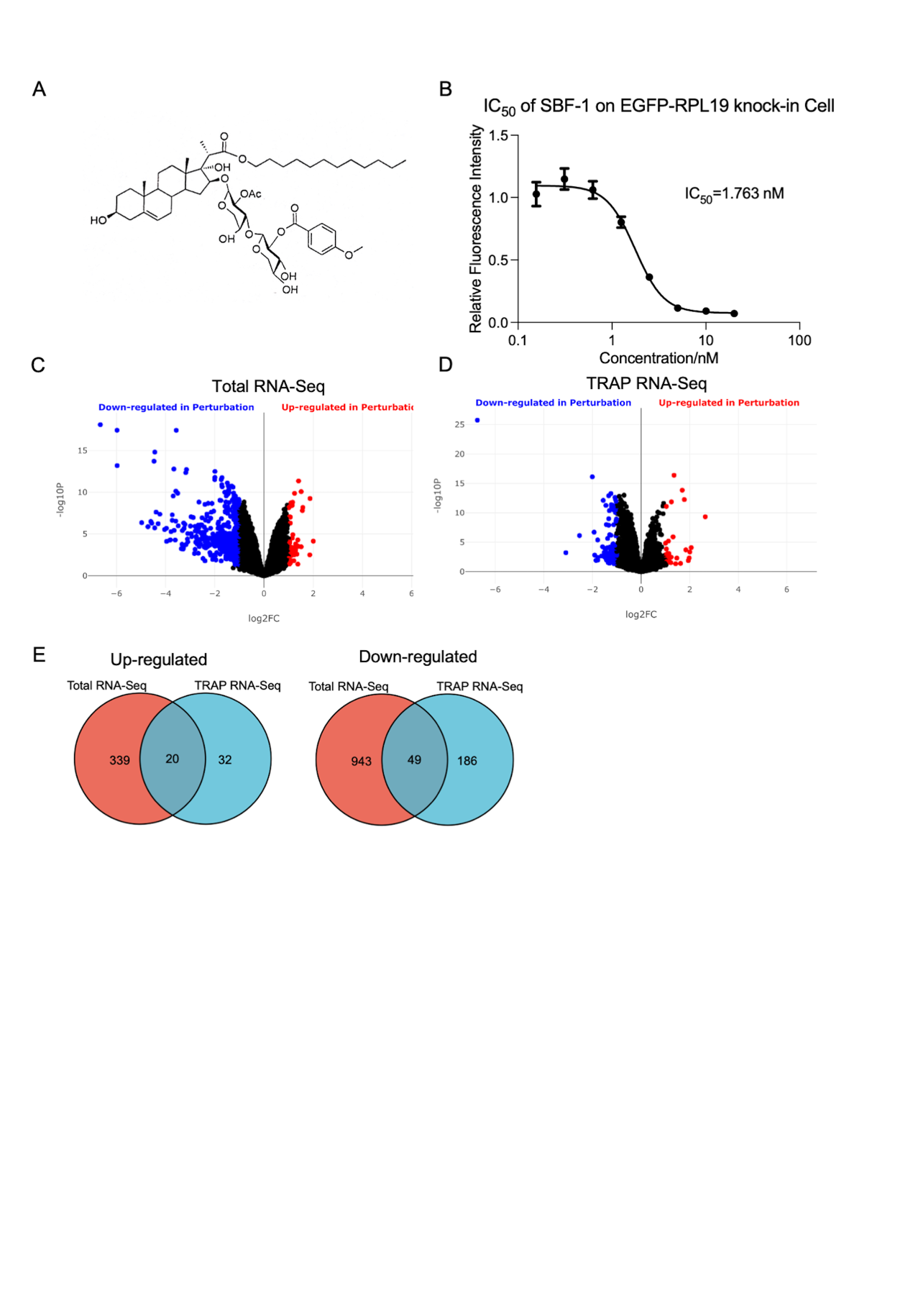


**Figure S3. RPL19-TRAP^KI^-Seq was employed for the target identification of SBF-1**

(A) The chemical structure of SBF-1.

(B) EGFP-RPL19 knock-in cells were treated with DMSO or SBF-1 at indicated concentrations for 72 hours. Cell growth was determined using CellTiter-Glo® Luminescent Cell Viability Assay.

(C and D) Volcano plot showing up- and down-regulated genes in total RNA (C) and TRAP RNA (D) after SBF-1 treatment.

(E) Venn diagram illustrating the number of up- and down-regulated genes in total RNA and TRAP RNA after SBF-1 treatment.
